# The Analysis of Inflammation-Related Proteins in a Cargo of Exosomes Derived from the Serum of Uveal Melanoma Patients Reveals Potential Biomarkers of Disease Progression

**DOI:** 10.3390/cancers13133334

**Published:** 2021-07-02

**Authors:** Joanna Patrycja Wróblewska, Michał Stefan Lach, Katarzyna Kulcenty, Łukasz Galus, Wiktoria Maria Suchorska, Daniel Rösel, Jan Brábek, Andrzej Marszałek

**Affiliations:** 1 Department of Oncologic Pathology and Prophylaxis, Poznan University of Medical Sciences, 61-866 Poznan, Poland; amars@ump.edu.pl; 2 Department of Tumor Pathology, Greater Poland Cancer Centre, 61-866 Poznan, Poland; 3 Department of Cell Biology, Faculty of Science, Charles University, BIOCEV, 25150 Vestec, Czech Republic; rosel@natur.cuni.cz (D.R.); jan.brabek@natur.cuni.cz (J.B.); 4 Radiobiology Lab, Department of Medical Physics, Greater Poland Cancer, 61-866 Poznan, Poland; michal.lach@wco.pl (M.S.L.); katarzyna.kulcenty@wco.pl (K.K.); wiktoria.suchorska@wco.pl (W.M.S.); 5 Department of Electroradiology, Poznan University of Medical Sciences, 61-866 Poznan, Poland; 6 Department of Orthopedics and Traumatology, Poznan University of Medical Sciences, 61-544 Poznan, Poland; 7 Department of Medical and Experimental Oncology, Heliodor Swiecicki University Hospital, Poznan University of Medical Sciences, 61-866 Poznan, Poland; lukasz_galus@wp.pl; 8 Department of Chemotherapy, Greater Poland Cancer Centre, 61-866 Poznan, Poland

**Keywords:** uveal melanoma, exosomes, inflammation-related proteins, biomarkers

## Abstract

**Simple Summary:**

Uveal melanoma (UM) is the most common intraocular tumour in adults with a poor prognosis and extremely high mortality rate due to the development of metastatic disease. Despite good knowledge of the histological and genetic background of metastases formation, there is still a lack of specific biomarkers which would allow early detection of UM progression. Due to their unique molecular cargo consisting of proteins and nucleic acids, exosomes have been widely studied as carriers of biomarkers for cancer development and progression. In this study, we analyzed the inflammation-related protein cargo of exosomes derived from the serum of primary and metastatic UM patients and healthy donors using multiplex immunoassay technology. We showed a significant correlation between the disease stage and the concentration of inflammation-related proteins from exosomal cargo. Based on the obtained results, we propose the panel of exosomal proteins for early detection of uveal melanoma progression into metastatic disease.

**Abstract:**

Background: Uveal melanoma (UM) is the most common intraocular tumour in adults with a poor prognosis and extremely high mortality rate due to the development of metastatic disease. However, despite relatively good knowledge about the histological and genetic risk factors for metastasis development, there is no specific biomarker that would allow early detection of UM progression. Recently, exosomes and their molecular cargo have been widely studied in the search for potential biomarkers in several cancers. The purpose of this study was to analyze the inflammation-related protein cargo of exosomes derived from the serum of primary and metastatic UM patients and healthy donors. Methods: The exosomes were isolated from the serum of primary and metastatic UM patients and healthy donors. Using multiplex immunoassay technology, we analyzed the concentration of 37 inflammation-related proteins in obtained exosomes. Results: The analysis of protein cargo showed several molecules related to inflammation, such as interferon-gamma, interleukin 2, 22 and 12(p40), Pentraxin-3, TNFSF13B and TNFSF8 which were significantly enriched in metastatic UM exosomes. We showed a significant correlation between the disease stage and the concentration of these inflammation-related proteins from exosomal cargo. Conclusions: Based on the obtained results, we propose the panel of exosomal proteins for early detection of uveal melanoma progression into metastatic disease.

## 1. Introduction

Uveal melanoma (UM) is the most common intraocular malignancy in adults, although it accounts for only 3–5% of all melanoma cases. The main primary site for this type of cancer is the choroid (~90%), while the remaining cases arise from the ciliary body (5–8%) and the iris (3–5%) [1]. Risk factors for developing UM include fair skin, light-coloured eyes, and preexisting conditions such as congenital ocular melanocytosis, melanocytoma of BAP1-tumor predisposition syndrome [2]. The ultraviolet (UV) radiation exposure—the main risk factor for cutaneous melanoma—has also been considered, but the comprehensive analysis of data from The Cancer Genome Atlas (TCGA) project showed a very low tumour mutation burden and lack of UV-related molecular signature in UM tumours [3]. Despite the relatively good response of primary UM to the treatment, which includes surgery and radio/chemotherapy, over 50% of patients will develop metastatic disease. The most common site of metastasis is the liver, where spread occurs almost exclusively via blood vessels. The relapse and spread of the disease are highly correlated with poor prognosis, which in consequence results in only 8% of patients with metastatic UM surviving 2 years [3,4]. Currently, no effective therapies to prevent the development and treatment of metastatic disease are available, except for early detection and management of primary tumours [5,6]. Histological and genetic landmarks related to poor prognosis and increased risk of developing a metastatic disease in UM tumours have been described and tightly correlates with tumour size and chromosome 3 monosomy [3,7]. Despite this knowledge, there is a lack of a simple and minimally invasive test for early detection of metastasis. The current recommendation for screening of disease progression includes imaging techniques such as computer tomography, magnetic resonance, positron emission tomography or ultrasonography, which do not always guarantee detection of small lesions. Several blood biomarkers have been proposed including circulating tumour cells [8,9,10], circulating tumour DNA [11], and microRNA [12,13,14] and proteins [15,16,17,18,19], but still, there is a lack of reliable, clinically approved biomarker for early detection of UM and its progression.

Recent discoveries regarding a distinct population of extracellular vesicles secreted by malignant cells, especially exosomes could bring a solution to this problem. They have been widely studied as carriers of potential biomarkers for cancer development and metastatic spread in several cancers but not UM [20,21,22,23]. Exosomes are the smallest fraction of extracellular vesicles, up to 150 nm, formed within the endosomal network and released from multivesicular bodies after fusion with the cellular membrane. Their molecular cargo includes a variety of active biomolecules, i.e., proteins, lipids, miRNA, lncRNA, mRNA and DNA, characteristic for parental cells, making them essential participants in cellular interactions, regulators of a broad variety of biological processes. The existence of UM-derived exosomes has been confirmed by observing their presence in the hepatic circulation system, vitreous humour, blood serum, and in vitro cell culture. The protein cargo was analyzed in exosomes, ectosomes derived from UM, and normal melanocyte cell lines [24,25]. These results showed that the protein cargo of UM-derived extracellular vesicles may have transforming potential and could contribute to the preparation of the metastatic niche. Moreover, the isolation of UM-specific tumour-derived exosomes (TEX) allowed their use as a biomarker of disease development and progression and in the future may contribute to the design of early diagnostic tests. However, no data were obtained for exosomes derived from UM patients.

This study aimed to determine and compare the composition of inflammation-related proteins in exosomes derived from the serum of UM patients diagnosed with either primary or metastatic disease and healthy donors. Our goal was to point out potential biomarkers for simple and minimally invasive screening for early detection of UM metastatic spread.

## 2. Materials and Methods

### 2.1. Study Group

The study group consisted of serum samples obtained from 20 uveal melanoma patients and 20 healthy donors, after informed consent. The study was approved by the Bioethics Committee of Poznan University of Medical Sciences, study number 114/18. The serum samples were obtained from UM diagnosed patients with primary tumour localized in choroid only, before surgery (in the case of patients with the primary tumour, *n* = 9) or before implementing the systemic treatment (in the case of patients with metastatic disease, *n* = 11). The patients were divided into “primary” or “metastatic” group based on an histopathological evaluation of the primary site of the tumour (diagnosis: Uveal melanoma) and by the confirmed presence/absence of distant (liver) metastasis. The patient with histopathological recognition of UM in the choroid and absence of liver metastasis in CT/PET scan was allocated to the “primary UM” group. The patient with histopathological confirmation of UM in the choroid and its metastasis in the site of the liver (fine needle biopsy followed by histopathological examination) were recognised as the “metastatic UM” group. The healthy donors’ group includes healthy females and males, age 30–60, corresponding to the time of uveal melanoma onset. Donors with previous or ongoing immunological disease or cancer, ongoing viral or bacterial infection, ongoing immunosuppressive therapy, and vaccination in the period of 3 months before blood collection were excluded from the study.

### 2.2. Exosomes Isolation

Exosomes were isolated from 5 mL of serum from UM patients and healthy donors. Firstly, the serum was centrifuged for 30 min at 500× *g*. Furthermore, to remove larger debris and apoptotic bodies the obtained supernatant was centrifuged for 45 min at 12,000× *g*. The supernatant was filtered using 0.2/0.8 µm Acrodisc^®^ PF syringe filters (Pall, Port Washington, NY, USA) and transferred to Amicon^®^ Ultra-15 Centrifugal Filter Unit with a 100 kDa cut-off (Merck KGaA, Darmstadt, Germany) for the enrichment of the exosomes fraction. The concentrated serum was diluted in a phosphate-buffered saline (PBS) (Biowest, Nuaillé, France) in 1:1 ratio, transferred to ultracentrifuge tubes and centrifuged for 90 min at 120,000× *g* (fixed angle rotor 70.1Ti, Beckman Coulter, Indianapolis, IN, USA). All centrifuge steps were performed at 4 °C. The obtained pellet was resuspended in a RIPA Lysis buffer containing the protease inhibitor cocktail (both obtained from Sigma-Aldrich, St. Louis, MO, USA) or PBS depending on further destination. The amount of exosomal proteins was measured using the Pierce™ BCA Protein Assay Kit (ThermoFisher Scientific, Waltham, MA, USA) and frozen at −80 °C for further analysis. The method of exosome isolation and validation comply with the Minimum Information Supporting Extracellular Vesicle Research (MISEV 2018) suggestions including the imaging with scanning electron microscopy, confirmation of the presence of positive markers for exosomes, and absence of protein related to cellular compartments [26].

### 2.3. Western Blot Analysis of Exosomes

For the confirmation of isolation of exosomal fraction, the Western blot analysis was performed. In addition, 20 µg of the exosomal protein was mixed with a Laemmli sample buffer and heated for 15 min at 95 °C. Then, the cooled samples were transferred to 4–15% gradient polyacrylamide gel Mini-PROTEAN^®^ TGX™ Precast Protein Gels (Bio-Rad, Hercules, CA, USA). After electrophoresis, the proteins were transferred to a polyvinylidene fluoride (PVDF) membrane (Bio-Rad, Hercules, CA, USA), which was blocked in 5% nonfat dry milk (Sigma-Aldrich, St. Louis, MO, USA) suspended in Tris buffered saline/Tween 20 (TBST) (Tris Base, Sodium chloride both obtained VWR Chemicals, Leuven, Belgium; Tween 20, POCh, Gliwice, Poland) for 2 h at room temperature. Then, the membrane was incubated overnight at 4 °C with a primary antibody against proteins related with: Exosomal origin—LAMP1, Alix, CD63; functional marker TGFβ1; a negative marker of exosomes—Calnexin (the dilutions and vendors are listed in Table 1). On the next day, the membrane was washed with TBST and underwent a 2 h incubation at room temperature with a secondary antibody conjugated with horseradish peroxidase (HRP) diluted in 5% non-fat dry milk in TBST. To detect the proteins of interest, a chemiluminescent reaction was performed using WesternBright™ Quantum (Advansta, Inc., San Jose, CA, USA). The exposition and documentation of the membrane were done using the ChemiDoc™ Touch Imaging System (Bio-Rad Laboratories, Hercules, CA, USA). As a control, 20 µg of cell lysate of cutaneous melanoma cell line MW-266-4 was used (kindly provided by Urszula Kaźmierczak Ph.D., Department of Cancer Immunology, Poznan University of Medical Sciences).

### 2.4. Scanning Electron Microscopy

To confirm the morphology and size of isolated exosomes, scanning electron microscopy (SEM) was performed. Briefly, 10 µg of isolated UM- or HD-exo was diluted in PBS, transferred on the double-adhesion tape previously affixed to the aluminium SEM mount (Structure Probe, Inc., West Chester, PA, USA) and left to dry. Then, the samples were coated with gold using the Balzers SCD 050 sputter coater (Oerlikon Balzers, Balzers, Liechtenstein). Pictures were taken using the Scanning Electron Microscope Evo 40 Series (Carl Zeiss SMT AG, Oberkochen, Germany).

### 2.5. Acetylcholinesterase Activity

For the measurement of acetylcholinesterase activity in derived exosomes, 10 µg of exosomal proteins were diluted in PBS to a final volume of 50 µL and added to the wells of a 96-well flat-bottom plate. Next, a 50 µL reaction mixture of 2.5 mM acetylcholine and 0.1 mM 5,5′-dithio-bis (2-nitrobenzoic acid) (DNTB) (both obtained from Sigma-Aldrich, St. Louis, MO, USA) was added to the wells and mixed. After 20 min of incubation at 37 °C, the absorbance of analyzed samples was measured at 405 nm using the plate reader Multiskan FC (Thermofisher, San Jose, CA, USA). As a blank control, we used the diluent (PBS).

### 2.6. Analysis of Protein Composition of Exosomes

The protein composition of exosomes from uveal melanoma patients and healthy donors were analyzed with the Bio-Plex 200 system (BioRad Laboratories, Hercules, CA, USA) as previously described [27]. Briefly, we used the Bio-Plex Pro™ Human Inflammation immunoassay (cat. no. #171AL001M), to analyze 37 inflammation-related proteins that may be involved in the inflammation process, initiation, and progression of cancer in one sample. The concentrations (pg/mL) of analyzed molecules were compared to the standard curve of serial dilutions of standard proteins. The analysis was performed according to the manufacturer’s instructions. Each sample was analyzed in technical duplicate.

### 2.7. Statistical Analysis

Statistical analyses were performed using the GraphPad Prism software program, v.8 (GraphPad Software, Inc., La Jolla, CA, USA). As the data (pg/mL) did not follow Gaussian distribution, we used the Mann–Whitney test or the Kruskal–Wallis test. In addition, *p* < 0.05 was considered to indicate a statistically significant difference. The means of technical duplicates were visualized as box plots (median and whiskers) presenting the concentration of cytokines (pg/mL). Whiskers were calculated using the Tukey method based on the GraphPad Prism software. Moreover, * *p* < 0.05, ** *p* < 0.01, *** *p* < 0.001, **** *p* > 0.0001: Based on the Mann–Whitney or Kruskal–Wallis test with Dunn’s post hoc multiple comparison test. Heatmaps displaying median concentrations of analyzed cytokines were prepared in GraphPad Prism. The cut-offs were adjusted empirically to visualize changes (if present) both at the top and bottom of the heatmap.

## 3. Results

### 3.1. Exosomes Derived from the Serum of Uveal Melanoma Patients

We have isolated the exosomes from individual or pooled serum samples collected from 20 patients diagnosed with primary or metastatic UM (UM-exo primary and UM-exo metastatic) and 20 healthy donors (HD-exo). The groups were selected for the study based on their initial diagnosis, tumour localization, metastasis status, and implemented therapy. The patients were defined as the “primary” group (*n* = 9) and included cases diagnosed with UM, without metastasis on the day of the enrollment to the study. The group acknowledged as “metastatic” represented the patients with detected metastasis on the day of the qualification for study. To ensure homogeneity of the studied group only patients with a tumour localized in the choroid were included. The blood was drawn before implementing any therapy including surgery or radiotherapy (the primary group), and chemo- or immunotherapy (the metastatic group).

**Figure 1:**
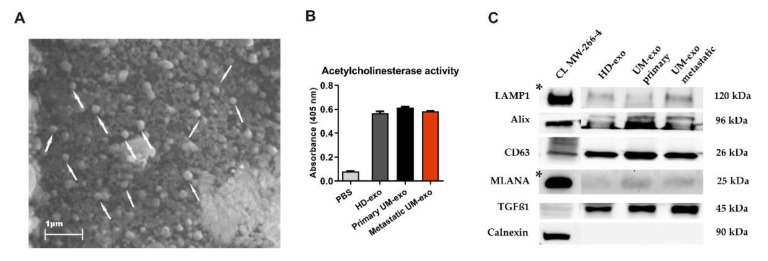
The characteristics of exosomes isolated from the serum of uveal melanoma patients with primary and metastatic disease (UM-exo primary and UM-exo metastatic) and healthy donors (HD-exo). The scanning electron microscopy (SEM) showed the proper shape and size (exosomes marked with white arrows) (**A**). The isolated exosomes indicate high levels of acetylcholinesterase activity. Data represent the mean ± SD from three technical replicates of absorbance readout at 405 nm from the pooled serum (**B**). The expression of proteins characteristic for small extracellular vesicles—endosomal origin (LAMP1, Alix, CD63), their protein cargo (TGFβ1), the tumour-origin (MLANA), and absence of organelle-specific proteins (Calnexin) was confirmed by Western blot. The band marked with * were obtained from the short-time exposure of the same membrane, due to differences in the protein expression level between the cell line lysate and exosomes (**C**). The uncropped Western Blot images can be found in Figure S1.

The exosomes were isolated using a serial ultracentrifugation approach and subjected to phenotype analysis. The scanning electron microscopy analysis revealed the presence of vesicle-like structures in the range below 200 nm (Figure 1A). Additionally, the presence of isolated exosomes was confirmed by the acetylcholinesterase activity (AChE) assay. The analysis showed higher levels of AChE activity in the analyzed samples in comparison with the diluent, but the level of absorbance was similar between the HD and UM groups (Figure 1B). The Western blot analysis of the molecular cargo of isolated exosomes showed the presence of Lysosomal associated membrane protein (LAMP1), Alix, and CD63, which confirms the endosomal origin of isolated vesicles. We also detected the transforming growth factor β1 (TGF-β1)—a molecule with pleiotropic functions. Surprisingly, we also detected a mild signal of melanocyte-specific protein (MLANA), which is recognized as one of the markers specific for melanoma. This confirms the enrichment of the exosomes population with tumour-derived ones. The purity of isolated extracellular vesicles was confirmed by negative staining for Calnexin, a protein related to the endoplasmic reticulum (Figure 1C).

### 3.2. The Immunomodulatory Protein Cargo of UM-exos

**Figure 2:**
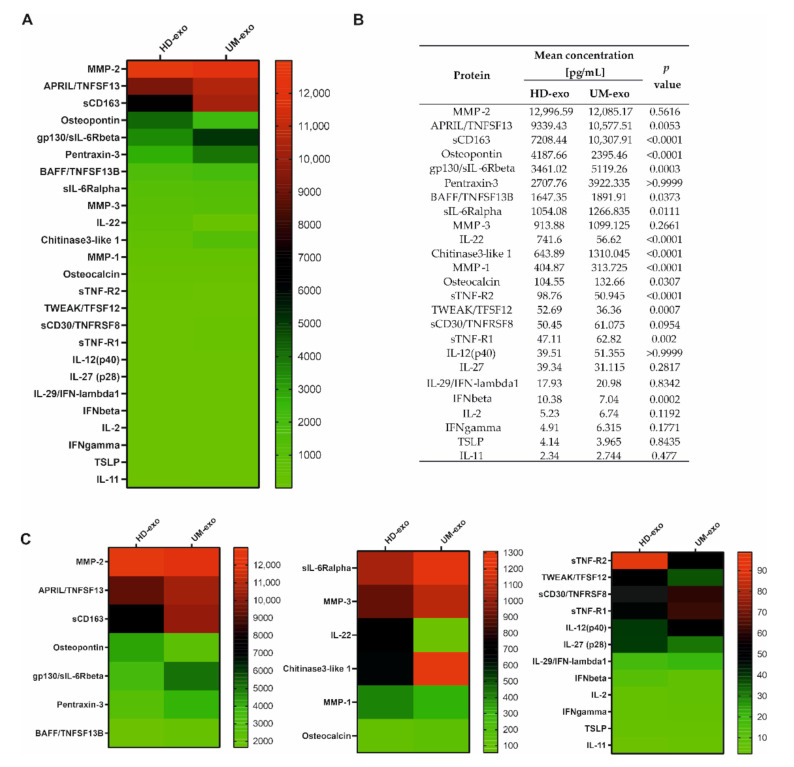
The general comparison of immunomodulatory protein in exosomal cargo in healthy donors and UM patients. All the concentrations are displayed in (pg/mL), only the proteins effectively detected are shown. (**A**) Heatmap representing mean concentrations of all the analyzed cytokines in HD-exo (*n* = 20) and UM-exo (*n* = 20) groups; (**B**) table showing mean concentrations and *p*-values indicating the statistical significance; (**C**) for better visualization of the differences in cytokine concentrations, the heatmap was divided into three panels with a distinct concentration range: 13,000–2000 pg/mL, 1400–100 pg/mL, 100–0 pg/mL. The *p*-value was calculated with the unpaired *t*-test or Mann-Whitney test.

To determine the immunoregulatory protein content in the exosomal cargo, we used the multiplex immunoassay technology containing 37 cytokines, chemokines, and growth factors related to the inflammation process. Altogether, we were able to confirm the presence of 25 cytokines, with concentrations ranging from around 2 to almost 13,000 pg/mL. A general comparison of the composition of exosomes derived from healthy donors and UM patients revealed significant differences among 14 proteins, with *p*-value ≤ 0.05. Moreover, most of the analyzed cytokines (17 of 25) had a higher concentration in UM patients-derived extracellular vesicles (Figure 2A–C). The increased concentration of eight proteins (A proliferation-inducing ligand/ tumor necrosis factor ligand superfamily member 13 (APRIL/TNFSF13), soluble CD163 (sCD163), Glycoprotein 130/soluble receptor for interleukin 6 beta (gp130/sIL-6Rbeta), B-cell activating factor / Tumor Necrosis Factor ligand superfamily member 13B (BAFF/TNFSF13B), soluble receptor of interleukin 6 alpha (sIL-6Rα), Chitinase 3—like 1, Osteocalcin, soluble Tumour Necrosis Factor receptor 1 (sTNF-R1)) was notified in UM-exosomes in comparison with HD-exo (Figure 2A–C). Moreover, among these 14 differentiating proteins, six of them (Osteopontin, interleukin 22 (IL-22), Matrix Metalloproteinase 1 (MMP-1), soluble Tumour Necrosis Factor receptor 1 (sTNF-R2), TNF-related weak inducer of apoptosis/Tumor Necrosis Factor ligand superfamily member 12 (TWEAK/TFSF12), interferon beta (IFN-β)) were downregulated in UM-exo versus exosomes derived from healthy donors. We did not observe significant changes between the cargo of UM-exo and HD-exo among the remaining 11 proteins effectively detected in the samples. These proteins include: Matrix Metalloproteinase 2 (MMP-2), Pentraxin-3, Matrix Metalloproteinase 3 (MMP-3), soluble CD30/ Tumor Necrosis Factor ligand superfamily member 8 (sCD30/TNFRSF8), interleukin 27 (IL-27), interleukin-12 subunit p40 (IL-12(p40)),interleukin 29/interferon lambda 1 (IL-29/IFN-λ1), interleukin 2 (IL-2), interferon gamma (IFNγ), thymic stromal lymphopoietin (TSLP), interleukin 11 (IL-11) (Figure 2A–C).

**Figure 3:**
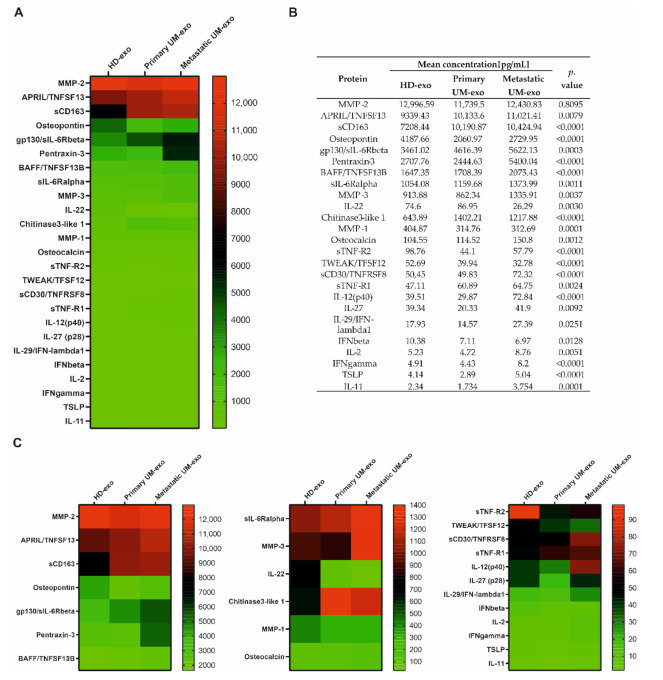
Immunomodulatory protein cargo of exosomes from healthy donors and UM patients. All the concentrations are displayed in (pg/mL), only the proteins effectively detected are shown. (**A**) Heatmap representing mean concentrations of all the analyzed cytokines in HD-exo, primary UM-exo and metastatic-exo groups; (**B**) table showing mean concentrations and *p*-values indicating the statistical significance; (**C**) to better visualize the differences in cytokine concentrations, the heatmap was divided into three panels with concentration range: 13,000–2000 pg/mL, 1400–100 pg/mL, 100–0 pg/mL. The *p*-value was calculated based on one-way ANOVA (data with normal distribution) or Kruskal–Wallis test (samples without normal distribution).

**Figure 4:**
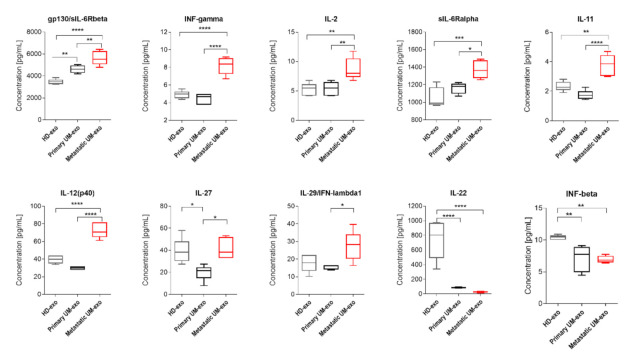
Box and whiskers plots present the concentration of interleukins and interferons detected in exosomes derived from healthy donors, primary and metastatic UM patients. Results are displayed as a median concentration in picograms per milliliter. The box and whiskers were calculated using the Tukey method. * *p* < 0.05, ** *p* < 0.01, *** *p* < 0.001, **** *p* > 0.0001: Based on one-way ANOVA or Kruskal–Wallis test with Dunn’s post hoc multiple comparison test.

**Figure 5:**
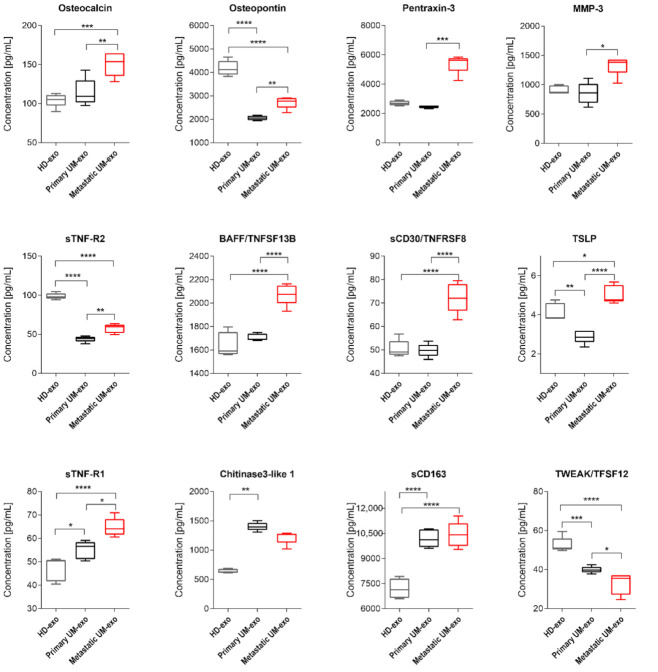
Box and whiskers plots presenting concentration of TNF superfamily, extracellular matrix and its modifying proteins in exosomes derived from healthy donors, primary and metastatic UM patients. Results are displayed as a median concentration in picograms per milliliter. Box and whiskers were calculated using the Tukey method. * *p* < 0.05, ** *p* < 0.01, *** *p* < 0.001, **** *p* > 0.0001: based on One-way ANOVA or Kruskal–Wallis test with Dunn’s post hoc multiple comparison test.

Furthermore, we evaluated the composition of the exosomes among three groups, which enabled the comparison of cytokines content not only between UM patients and healthy donors but also primary (*n* = 9) and metastatic UM (*n* = 11). We showed that most of the detected cytokines and growth factors (16 out of 25) were upregulated in exosomes derived from metastatic UM patients (Figure 3, Figure 4 and Figure 5).

The amount of gp130/sIL-6Rbeta was the lowest in exosomes derived from the healthy donor serum in comparison with primary and metastatic UM-exo. In contrast, the highest content of that protein was identified in the metastatic group (Figure 4). Similarly, the other detected proteins (INF-γ, IL-2, sIL-6Rα, IL-11, IL-12p40) have also been exhibiting the highest concentration in exosomes derived from metastatic UM patients. In the aforementioned concentration of proteins, we did not observe the significant changes between the exosomes derived from healthy donor groups and primary UM patients (Figure 4). In the case of IL-27, its concentration was higher in exosomes derived from the healthy donors pooled serum in comparison with primary UM-exo (Figure 4). A similar trend was observed in exosomes derived from metastatic UM, where the IL-27 concentration was elevated in comparison with the primary UM-exo. The analysis of IL-29/IFN-λ1 in exosomes cargo revealed that there were no differences between its concentration in exosomes derived from healthy donors and primary or metastatic patients. Metastatic UM-exo exhibited increased IL-29 content but this increase was statistically significant only when compared to primary UM-derived exosomes. Meanwhile, the content of IL-22 and IFN-beta in an exosomal cargo of both patients groups were decreased in comparison with the level of those proteins in HD-exo. Moreover, between primary and metastatic groups there was a lack of differences in their concentration (Figure 4).

Among detected proteins in exosomal cargo, we were able to distinguish those related to extracellular matrix proteins and their modifiers, and the TNF family (Figure 5). Pentraxin-3 concentration was not different between HD-exo and primary UM-exo or metastatic UM-exo, but between UM groups the increased content of that protein was observed in the metastatic group compared to the primary one (Figure 5). Osteocalcin content in exosomes has an increasing trend in presented groups, with the highest concentration in metastatic UM-exo when compared to both healthy donors and primary UM groups. However, between HD-exo and primary UM-exo differences were non-significant (Figure 5). Meanwhile, Osteopontin protein levels from both UM groups were notably lower than in exosomes from the healthy donors’ group, but its concentration was significantly higher in metastatic UM than in primary UM (Figure 5). We also detected the presence of extracellular matrix modifying proteins (MMP1, 2, 3) with MMP2 being the most abundant protein (Figure 2, Figure 3 and Figure 5). Metastatic UM-exo exhibited increased MMP3 concentration, but this increase was statistically significant only when compared to primary UM-derived exosomes (Figure 5). The exosomal cargo was also loaded with diverse proteins belonging to tumor necrosis factor (TNF) family. The sTNF-R2 expression was decreased in UM-derived exosomes in comparison with HD-exo. However, among UM group, the exosomes derived from metastatic patients exhibited an increased amount of that protein in comparison with a cargo of primary UM-derived exosomes (Figure 5). The adverse trend was notified BAFF/TNFSF13B, where its content was the highest in the metastatic UM exosomes. Nevertheless, in the HD-exo and primary UM-exo we did not observe the differences (Figure 5). Similar trend and differences in the studied groups were observed in the sCD30/TNFRSF8 as notified in the BAFF/TNFSF13B (Figure 5). In the TSLP, again the highest content was observed in the UM exosomes derived from metastatic patients. Surprisingly, the primary UM patients had the lowest amount of TSLP in the exosomal cargo in comparison with the studied groups (Figure 5). We showed that exosomes from metastatic UM contain a higher load of BAFF/TNFSF13B, and TSLP than from the primary UM or HD group. Similarly, to the previously studied protein, the highest concentration of sTNF-R1 was notified among exosomes obtained from the serum of metastatic patients. Moreover, the concentration of sTNF-R1 was also notably higher in primary UM than in HD-exos. It is worth mentioning, the expression of Osteopontin, sTNF-R2, TWEAK/TNFSF12 was significantly lower in exosomes derived from both primary and metastatic UM patients when compared to healthy donors. TWEAK/TNFSF12 was also the only protein with a concentration significantly lower in metastatic UM compared to primary UM-exos. The amount of Chitinase3-like 1 was higher in the UM-exo, but the statistical significance was achieved only in primary patients. In the case of soluble CD163 (sCD163) we observed a significantly higher level of that protein in both UM-exos compared to HD-exo, but in terms of comparison of primary and metastatic groups, we did not notify the differences (Figure 5).

Taken together, our results point out important differences in the molecular cargo of exosomes derived from the serum of patients with primary or metastatic UM and healthy donors. Significant differences in inflammation-related protein concentration may be used as potential biomarkers for the early detection of disease progression.

## 4. Discussion

The UM despite its known biology is still challenging malignancy in oncological ophthalmology, mainly caused by the poor prognosis related to the development of metastatic disease. A secretion of specialized extracellular vesicles via many cancers has revealed their role in the formation of metastatic niche, which is a complicated and complex process involving modification of target microenvironment and immune cells. In the case of UM patients, it is not a well-described phenomena. That is why in the presented study, we decided to estimate the cargo of the UM exosomes derived from the serum of patients with primary and metastatic disease to find specific proteins responsible for the spread of UM cells, which possibly could be used as a predictive biomarker. The existence of UM-specific exosomes has already been confirmed in several studies focusing on their protein content in vitro (in cell culture supernatant) and in vivo (in the vitreous humour, blood serum, and hepatic circulation system) [24,25,28,29,30]. However, their role as potential carriers of biomarkers for early detection of UM progression has not been studied. Angi et al. have analyzed the secretome of low- and high-risk UM cell lines derived from primary tumours and they showed their involvement in signalling pathways associated with remodelling of extracellular matrix, cell adhesion, activation of hepatic fibrosis/hepatic stellate cell, EIF2, Rho and IGF-1 signalling, and cancer cell migration/invasion [31]. Moreover, Zhao et al. showed that over 60% of detected proteins in vitreous humour derived from UM patients was associated with “extracellular exosomes” GO term. Further analysis of vitreous samples showed the presence of a vesicles’ population with size and morphology consistent with exosomes [28]. The proteome analysis of UM Mel202 cells-derived ectosomes, extracellular vesicles with size ranging from 50 nm to over 1 µm, identified 949 proteins, which most of them were related to cytosolic or membrane origin. Among them also 66 cancer-related proteins associated with cell proliferation/apoptosis, cell invasion and metastasis, cancer cell metabolism, drug resistance and heat-shock proteins were identified [25]. The most recent study presented by Tsering’ group has focused on the proteome of exosomes derived from 5 UM cell lines and normal choroidal melanocytes. They found out that their content is associated with cell-cell and focal adhesion, endocytosis, and PIK3-Akt signalling pathway. Moreover, the exosome-treated BRCA1-deficient fibroblast was shown to acquire a more CAF-like phenotype, with increased proliferation, migration, and invasion ability and this was related to enhanced tumour cells growth in vivo [24].

In our study, we focused on the analysis of the inflammation-related proteins cargo of exosomes derived from the serum of healthy donors and UM patients with primary and metastatic disease using the bead-based immunoassay and pointing out potential proteins related to the disease progression and metastasis occurrence. The inflammatory process plays a critical role in cancer development, progression, regulation of anti-cancer immune response, and recruiting tumour-promoting immune cells [32,33]. The concentrations of detected inflammation-related interleukins, interferons, proteins from the TNF superfamily, and their soluble receptors were significantly changed in most of the analyzed proteins in exosomes isolated from UM patients in comparison with these from healthy donors.

Interleukins are a diverse, multifunctional group of proteins that facilitate communication between various immune cells, control inflammation processes, and regulate cellular and humoral immunity [34]. Their roles in cancer biology have been thoroughly studied and they were proved to have both pro- and anticancer properties or pleiotropic effects depending on the tumour environment [35]. We demonstrated a significant upregulation of IL-2, IL-11, IL-12(p40), IL-27, and soluble receptors of IL-6 in exosomes derived from metastatic UM patients. Up to date, there is limited data on the role of these cytokines in UM development and progression but their presence has been confirmed in the vitreous humour of UM patients. However, there is a lack of reports regarding the presence of mentioned cytokines in UM-derived exosomes [36,37,38]. IL-2 has been proven to act as a potent activator of proliferation, differentiation, as well as cytotoxicity of T cells and NK cells. Moreover, it has been used as a promising agent in immunotherapy for cutaneous melanoma and renal cancer. The use of IL-2 has also been proposed to treat UM in combination with adoptive T cell transfer, where after lymphodepleting chemotherapy, the IL-2 is administered to the patients to boost T cell proliferation [39]. Another interleukin, IL-11, is a member of the IL-6 family, with pleiotropic actions participating in JAK/STAT signalling pathway [40]. As shown by Tao et al. in lung cancer, IL-11 has a potential role in modifying the tumour microenvironment and response to treatment, by recruiting the CAFs to and facilitating chemoresistance of cancer cells [41]. Moreover, IL-11 was shown to be responsible for poor prognosis in several cancers (including melanoma) correlated with staging and development of metastases in breast cancer, which correlates with our findings [42,43]. A similar correlation was found out during the estimation of serum level of IL-12(p40) in melanoma, but not directly in exosomes [44]. This cytokine acts as a chemoattractant and facilitates macrophage polarization into M1 proinflammatory phenotype, stimulating the interferon gamma (IFN-γ) production [45]. Another protein with double function pro-and anti-tumour functionality is IL-27, which belongs to the IL-12 family and is known for its ability to inhibit angiogenesis and activate NK cells [46]. Similarly to our studies the serum level of IL-27 did not correspond with metastatic disease as it was shown in the example of melanoma. However, there is a lack of data about their role in exosomal cargo [47,48,49], but their high expression, in general, is one of the markers of response to immunotherapy. Although we did not detect the IL-6 in the UM patient-derived exosomes, we detected its soluble receptors, which may alter the sensitivity to IL-6 stimulation of cells exposed to these exosomes. IL-6 is a pro-inflammatory cytokine typically produced by T lymphocytes and macrophages. Uveal melanoma cells and tumor-associated macrophages have also been shown to produce IL-6. The high concentration of IL-6 compared to healthy individuals has been detected in vitreous humour of UM patients, where it has been positively correlated with the tumour size, infiltration of T cells, and macrophages and disease progression [36,37,38].

The interferons (IFNs) are cytokines with pleiotropic properties, which can have beneficial effects in various tumour models. IFN-alpha and -beta are a potent stimulator of immune responses and tumour regression by regulation of cell proliferation, differentiation, and apoptosis and by activating NK cells and macrophages. The anti-tumour potential of IFN-alpha has been used in uveal melanoma therapy, however, with limited success [50,51]. In the cancer microenvironment, tumour-infiltrating lymphocytes and tumour cells mainly produce IFN-gamma. IFN-gamma has been detected in high concentration in the vitreous humour and blood serum of uveal melanoma patients, where it correlates with advanced disease stage and forming of metastasis [36,37]. In our study, we detected a significantly higher concentration of IFN-gamma and -lambda, and a lower concentration of IFN-beta in metastatic UM exosomes, which is consistent with previously published data and biological function of those proteins [52,53]. Moreover, the significant difference in concentration of IFN-gamma between metastatic UM and other exosomes suggests that this protein may be used as one of the potential markers for early detection of disease progression.

Tumour necrosis factor (TNF) and tumour necrosis factor receptor (TNFR) superfamilies’ are pro-inflammatory proteins involved in the regulation of diverse cell functions including immune response and inflammation, proliferation, differentiation, and apoptosis [54]. Recent studies are pointing out the pleiotropic role of TNF-alpha in cancer development and progression [55]. Apart from the well-described antitumor activity, it has been shown that in melanoma, colorectal carcinoma, the breast cancer TNF-alpha increases the cells’ tumorigenic properties [56,57,58,59]. Damento et al. observed several cases of uveal melanoma development in patients subjected to treatment with TNF inhibitors [54,60]. However, previous studies showed the presence of TNF-alpha in the vitreous humour of UM patients, where it correlates with tumour prominence and size [36,38]. No data are describing the role of exosome derived TNF and TNFR superfamily proteins in UM pathology. In our study, we detected the presence of TNF receptors superfamily: The sTNF-R1, sTNF-R2, TNFRSF8, and TNFRSF13B to be significantly upregulated in metastatic UM patient-derived exosomes when compared to primary patients, except sTNF-R2, to the HD group.

In our study, we also showed that UM- and HD derived exosomes contain proteins of the extracellular matrix milieu: Extracellular matrix modifying proteins—matrix metalloproteinases (MMP) 1, 2, and 3, Pentraxin-3, Osteopontin and Osteocalcin. The most abundant protein detected in exosomes was MMP2, however, there were no differences in its concentration between HD- and UM-exos. Of the detected metalloproteinases, the only concentration of MMP3 was significantly higher in metastatic UM-exos, which is consistent with its role in mediating cancer progression. It has been shown by Taha’s group that the knockout of MMP3 not only impairs cancer cell’s tumorigenic potential but also reduces the stability of cancer-derived extracellular vesicles [61]. Another detected modulator of ECM and better known as protein-related with acute-phase protein, Pentraxin-3, has been implicated in cancer progression, by modulating angiogenesis, metastatic spread, and cancer immune response [62,63]. Significantly high concentrations detected in metastatic UM derived exosomes compared to primary UM not only suggest an important role of Pentraxin-3 in UM metastatic spread but also may be used as a promising marker for early detection of disease progression. Recent data presented by the Rathore group suggest its role in the promotion of migration of melanoma cells through the TLR4/NF-κB signalling pathway [63]. To our surprise, the concentration of Osteopontin, another potent mediator of cancer metastasis involved in modulation of immune response and tumour stroma, was significantly lower in UM-derived exosomes compared to healthy donors. These findings contradict the literature data that shows an elevated level of Osteopontin in several cancers and its correlation with disease stage [64,65,66]. Several studies showed that the serum of patients with metastatic UM contains a significantly higher concentration of Osteopontin than the serum from primary patients or healthy donors [16,67]. A recent study by Song et al. also showed higher serum level of this protein in UM patients compared to healthy donors, however, no changes were observed between UM patients with the ongoing disease and those disease-free for 5 years [53,68]. Previous studies were focused on the serum, not the exosomal protein level, which may explain differences between literature data and those obtained in this study, as there is a possibility that in UM, Osteopontin is not secreted into extracellular vesicles.

In our previous study, we reported the possible use of miRNAs differentially expressed in UM tumours as biomarkers for the assessment of metastatic risk in uveal melanoma patients [69]. In this study, we present the first analysis of inflammatory-related protein cargo of exosomes derived from the serum of healthy donors and uveal melanoma patients with primary and metastatic disease. Based on these results, we can also point out several UM exosome-derived proteins, which can be utilized as promising markers for early detection of tumour development and progression into metastatic disease. The significant differences in the concentration of IFN-gamma, IL-2, IL-11, IL-12(p40), Pentraxin-3, TNFSF13B, and TNFSF8 between primary and metastatic uveal melanoma exosomes show the potential to utilize this panel of proteins for early detection of UM progression.

Our study has also a few limitations. We are aware, that during the isolation of exosomes a coprecipitation of soluble proteins and lipoproteins may occur, regardless of the methods used. This phenomenon may raise questions about the specificity of analyzed proteins, as they are present in blood in both a soluble and an exosome-encapsulated form [70]. Based on the published data, we decided to use the ultracentrifugation methods, with the best ratio for cost-effectiveness, efficiency, and purity of obtained exosomes [71]. The metastasis process changes the amount of secreted proteins, but we can hypothesize, that the ratio between soluble proteins and those encapsulated in exosomes might stay unchanged. However, to confirm this hypothesis, further studies are required. Our study presents preliminary results, with several proteins that may have the potential to point out the patients with UM progression. Their true usefulness in routine clinical diagnostic procedures is yet to be established and require further studies on a larger group of patients with long-term follow-up. Taken together, our results show that the proteins loaded into UM-exos have the potential to modify important biological processes involved in cancer development and metastatic spread. We propose a panel of exosomal proteins that may serve as potential biomarkers for minimally invasive detection of uveal melanoma progression into metastatic disease.

## 5. Conclusions

Early detection of metastatic uveal melanoma is still a major concern in the management of UM patients. Despite relatively good knowledge on the risk factors of disease progression, there is still a lack of simple and minimally invasive tests for its detection, which results in the extremely low survival rate of patients with metastatic disease. The exosomes have been widely described as carriers of possible biomarkers for cancer progression. In this study, we present the analysis and comparison of inflammatory-related protein cargo of exosomes derived from the blood serum of uveal melanoma patients with primary and metastatic disease and healthy donors. We detected a panel of differentially expressed proteins, which held great potential in discriminating primary and metastatic UM patients. We demonstrated a significant upregulation of IL-2, IL-11, IL-12(p40), IL-27, soluble receptors of IL-6, IFN-gamma and -lambda, TNF receptors superfamily: The sTNF-R1, sTNF-R2, TNFRSF8 and TNFRSF13B, and Pentraxin in exosomes derived from metastatic UM patients. Based on the obtained results, we propose the panel of exosomal proteins: IFN-gamma, IL-2, IL-11, IL-12(p40), Pentraxin-3, TNFSF13B and TNFSF8, which shows the potential to utilize this panel of proteins for early detection of UM progression. The results obtained in this study require further investigation. However, we believe that the described molecules have the potential to be utilized as markers for determining the risk and early detection of disease progression.

## Figures and Tables

**Table 1:** Antibodies and dilutions used in Western blot.

Antibody	Vendor	Dilution	Host
Calnexin	Santa Cruz, Dallas, TX, USA	1:500	Mouse
CD63	Santa Cruz, Dallas, TX, USA	1:500	Rabbit
LAMP1	Santa Cruz, Dallas, TX, USA	1:500	Mouse
ALIX	Santa Cruz, Dallas, TX, USA	1:500	Mouse
MELAN-A/MART-1	Cell Signaling Technology, Leiden, The Netherlands	1:250	Rabbit
TGFβ1	Cell Signaling Technology, Leiden, The Netherlands	1:1000	Rabbit
Anti—rabbit IgG-HRP	Cell Signaling Technology, Leiden, The Netherlands	1:2000	Goat
Anti—mouse IgG-HRP	Cell Signaling Technology, Leiden, The Netherlands	1:2000	Horse

CD63—cluster of differentiation 63 (tetraspanins); LAMP1—lysosome-associated membrane glycoprotein 1; ALIX—ALG-2-interacting protein X, MELAN-A/MART-1—melanoma antigen recognized by T cells 1; TGFβ1—transforming growth factor β1.

## Data Availability

All relevant data were presented in the study.

## Supplementary Materials

The following are available online at https://www.mdpi.com/article/10.3390/cancers13133334/s1. Figure S1. Uncropped Western Blot images.
