# Supplementary material for: The Analysis of Inflammation-Related Proteins in a Cargo of Exosomes Derived from the Serum of Uveal Melanoma Patients Reveals Potential Biomarkers of Disease Progression
Source: Cancers (Basel). 2021 Jul 2;13(13):3334. doi: 10.3390/cancers13133334 (PMC8268237; doi:10.3390/cancers13133334)
Supplement: Supplementary file 1 [file cancers-13-03334-s001.zip › cancers-1271146-supplementary.pdf]

Supplementary Materials: The Analysis of Inflammation-Related Proteins in a Cargo of Exosomes Derived from the Serum of Uveal Melanoma Patients Reveals Potential Biomarkers of Disease Progression

Joanna Patrycja Wróblewska, Michał Stefan Lach, Katarzyna Kulcenty, Łukasz Galus, Wiktoria Maria Suchorska, Daniel Rösel, Jan Brábek and Andrzej Marszałek

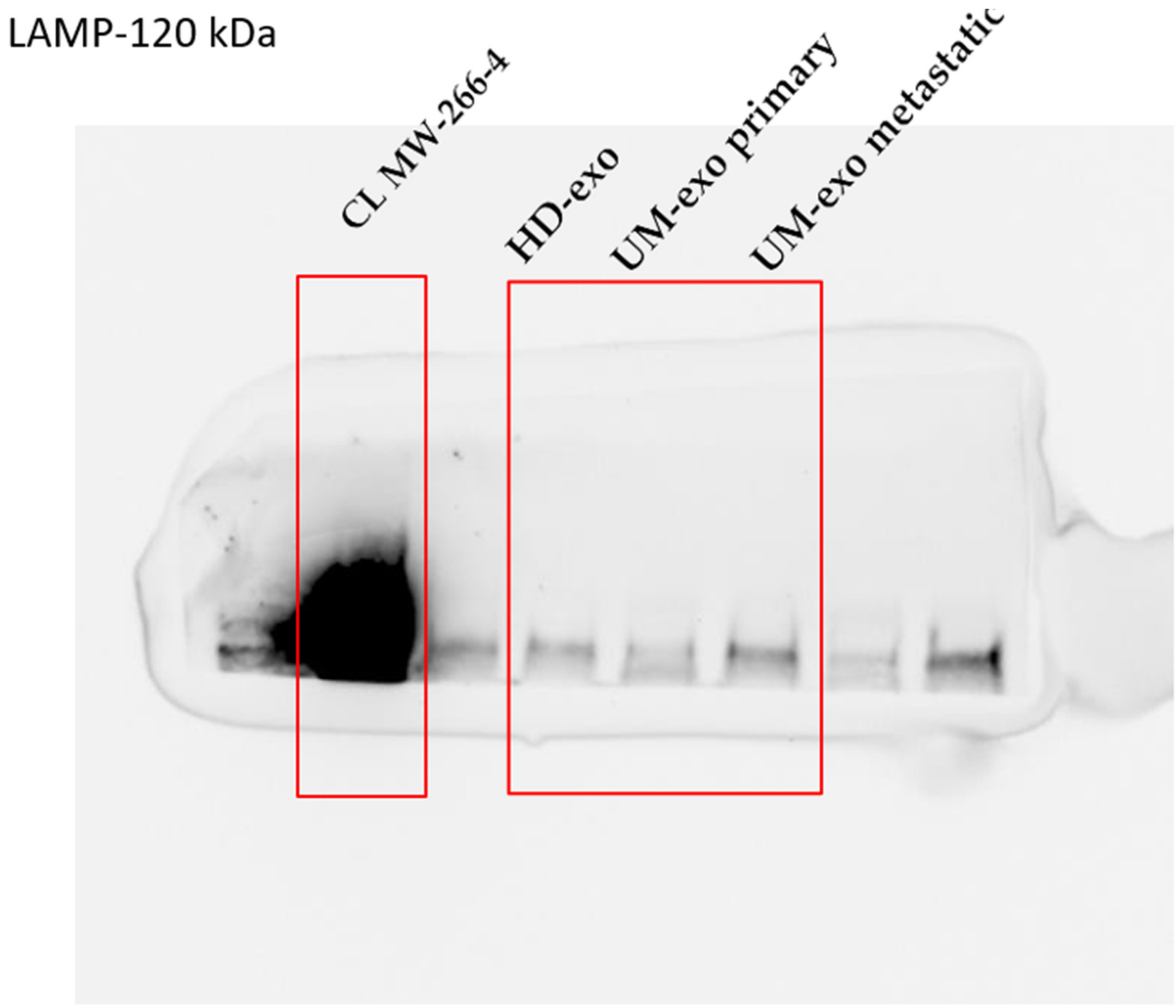

Alix – 96 kDa

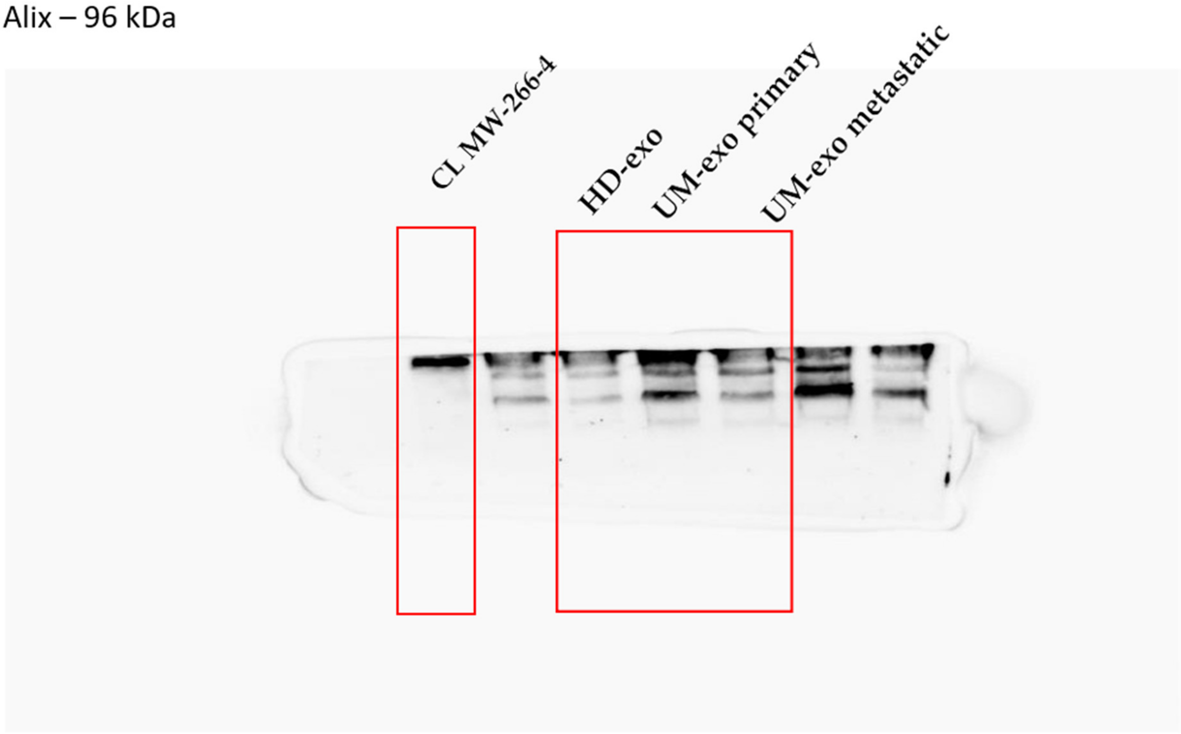

CD63- 26 kDa

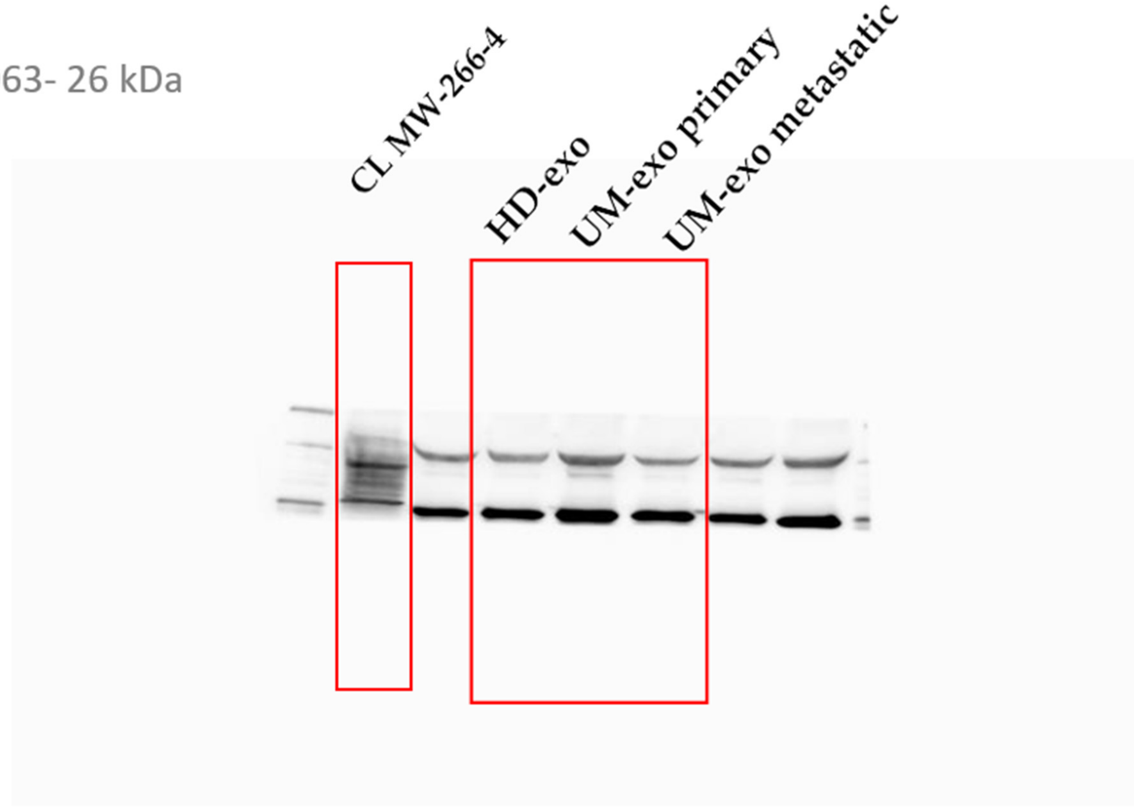

MLANA – 25 kDa

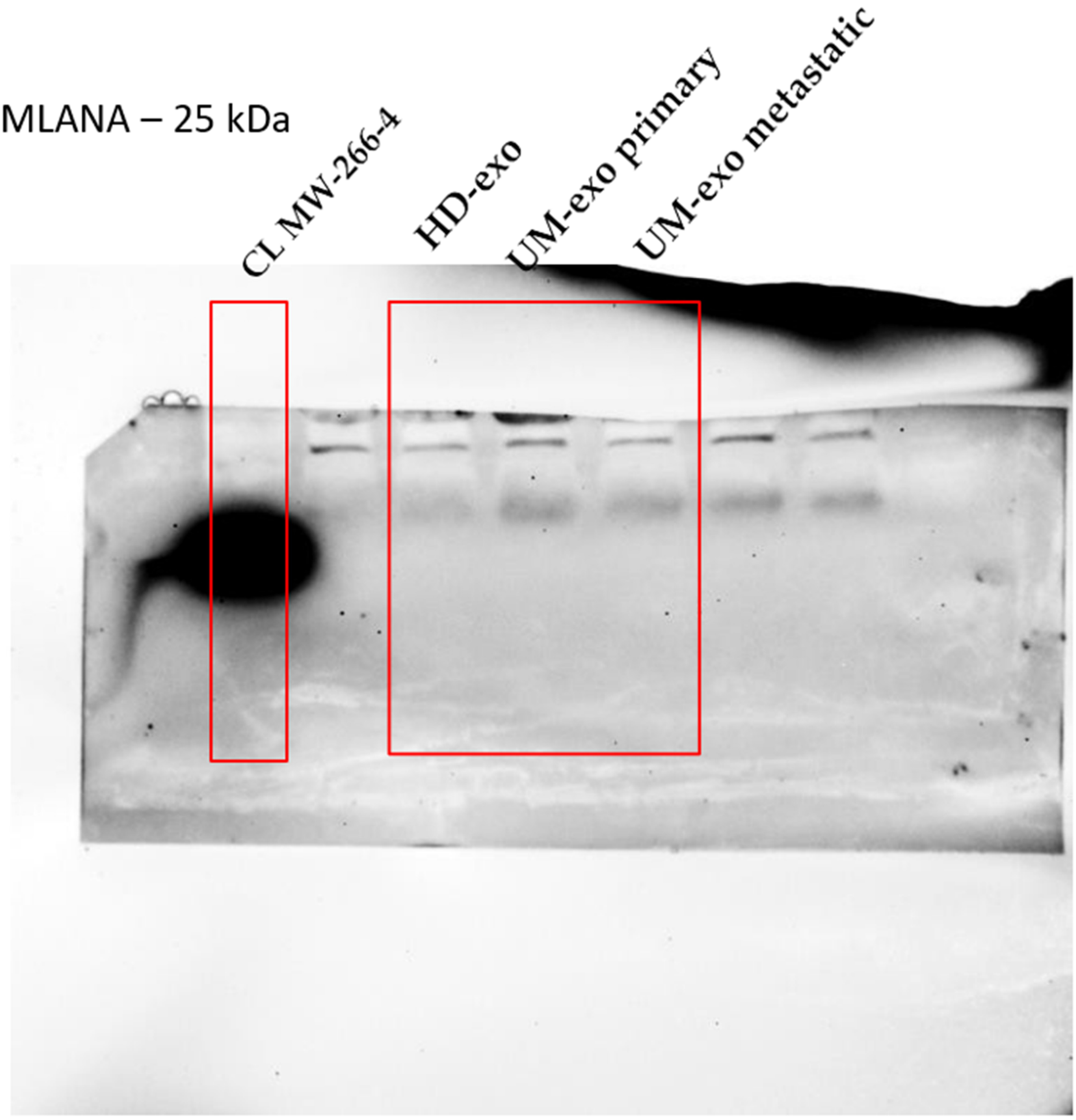

TGFβ1 – 45 kDa

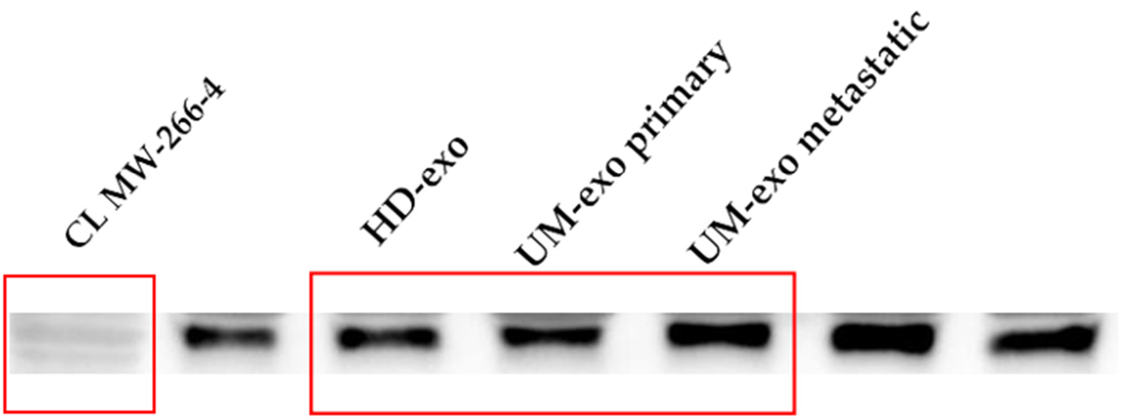

Calnexin – 90 kDa

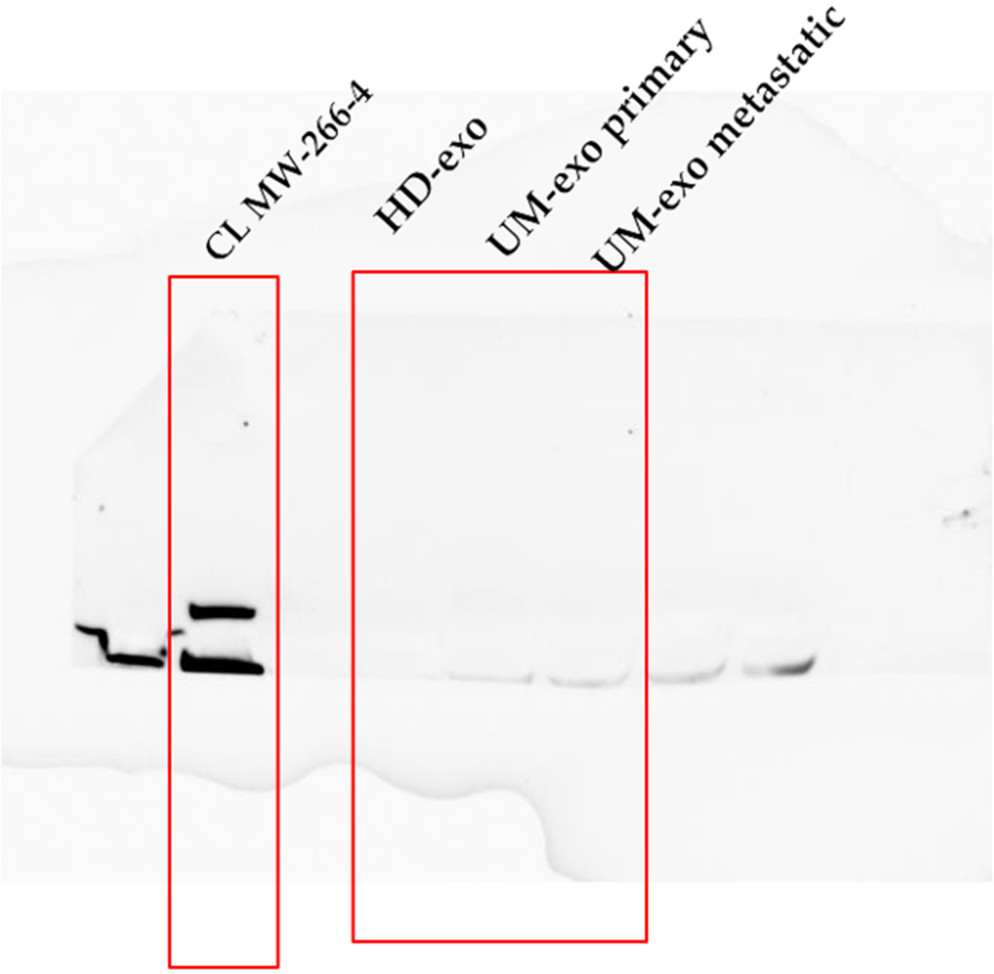

Figure S1. Uncropped Western Blot images.
